# Genetic susceptibility and environmental factors in dementia with Lewy bodies: Converging pathogenic mechanisms

**DOI:** 10.3389/fneur.2026.1825397

**Published:** 2026-04-24

**Authors:** Waleska Berrios, Angel Golimstok, María Cecilia Fernández

**Affiliations:** 1 Department of Neurology, Hospital Italiano de Buenos Aires, Buenos Aires, Argentina; 2 Universidad Hospital Italiano de Buenos Aires, Buenos Aires, Argentina

**Keywords:** air pollution, copathologies, environmental risk factors, Lewy body dementia, Lewy body disease, synucleinopathies

## Abstract

Dementia with Lewy bodies (DLB) is a heterogeneous neurodegenerative disorder characterized by cognitive decline, neuropsychiatric symptoms, and parkinsonism, with *α*-synuclein pathology as a central hallmark. Despite growing recognition of its clinical and biological complexity, the determinants underlying susceptibility to DLB remain incompletely defined and are frequently extrapolated from Parkinson’s disease. This review integrates recent evidence on genetic susceptibility and environmental and metabolic factors implicated in DLB, with emphasis on the biological mechanisms that may link these domains. Genetic studies support a moderate heritability and identify key risk loci, including *APOE*, *GBA*, and *SNCA*, which delineate biologically distinct subgroups and influence lipid metabolism, lysosomal function, mitochondrial quality control, and neuroinflammatory responses, with additional modulation by epigenetic and sex-specific factors. Environmental exposures, including pesticides, air pollution, heavy metals, and endocrine-disrupting chemicals, are associated with *α*-synuclein aggregation, mitochondrial dysfunction, neuroinflammation, and disruption of the gut–brain axis, largely based on experimental and observational evidence. Rather than defining a unified pathogenic cascade, current data support a framework in which genetic background constrains biological vulnerability, while environmental and metabolic exposures modulate disease expression and heterogeneity in DLB.

## 1 Introduction

Dementia with Lewy bodies (DLB) is the second most common cause of neurodegenerative dementia after Alzheimer’s disease (AD). It is characterized by the combination of progressive cognitive impairment, neuropsychiatric symptoms, and spontaneous parkinsonism, resulting in a substantial impact on quality of life and healthcare burden (1). From a neuropathological perspective, DLB belongs to the spectrum of synucleinopathies and is defined by the pathological accumulation of misfolded *α*-synuclein in Lewy bodies and Lewy neurites, with cortical and subcortical involvement affecting structures such as the brainstem, limbic system, and neocortex (1).

DLB exhibits marked clinical, neuropathological, and biological heterogeneity. Recent reviews highlight the existence of distinct pathological trajectories, with variability in the initial topography and propagation of *α*-synuclein pathology (“brain-first” versus “body-first” patterns), as well as a high prevalence of copathologies, particularly AD, that significantly influence clinical presentation and rate of disease progression (1–3).

*α*-Synuclein occupies a central role in the pathophysiology of DLB. Evidence indicates that this protein fulfills essential physiological functions, including regulation of presynaptic vesicle trafficking, membrane homeostasis, and modulation of mitochondrial function. Moreover, *α*-synuclein has been shown to play a critical role in extracerebral systems, including the immune, hematopoietic, and gastrointestinal systems, supporting a broader view of synucleinopathies as systemic disorders (4). This framework supports a bimodal pathophysiological model in which toxicity related to *α*-synuclein aggregation coexists with the loss of its normal physiological functions (“synucleinopenia”), with potential consequences for neuroinflammation, immune dysfunction, and gut–brain axis communication, mechanisms increasingly implicated in DLB (4).

In neuropathological contexts, the term Lewy body disease (LBD) is used to denote the spectrum of neurodegenerative disorders that includes Parkinson’s disease (PD), Parkinson’s disease dementia (PDD), and DLB, reflecting their shared substrate of misfolded *α*-synuclein pathology in the central and peripheral nervous system (5, 6). In clinical contexts, Lewy body dementia is an umbrella term encompassing DLB and PDD (1).

PD is primarily characterized by a motor syndrome including bradykinesia, rigidity, and tremor, with cognitive impairment typically emerging later in the disease course (7). In contrast, DLB is defined by early cognitive impairment accompanied by core features such as cognitive fluctuations, recurrent visual hallucinations, REM sleep behavior disorder, and parkinsonism. Operationally, dementia occurring before or within 1 year of parkinsonism supports a diagnosis of DLB, whereas dementia developing in the setting of established PD is classified as PDD (1). Neuropathologically, while both share *α*-synuclein aggregation, differences in the distribution and burden of pathology, as well as the frequency of coexisting Alzheimer-type changes, contribute to their clinical distinction (5, 8). Although these entities may represent distinct expressions within the clinical and neuropathological spectrum, each has been well characterized; therefore, maintaining this distinction remains relevant in clinical practice and research.

From a clinical and epidemiological perspective, a recent systematic review identified limited evidence regarding risk factors and predictors associated with the development of DLB. The most consistently reported factors include early clinical manifestations, such as mild cognitive impairment and REM sleep behavior disorder, as well as a history of herpes simplex virus infection and adult attention-deficit/hyperactivity disorder (9). In parallel, DLB-specific genomic evidence supports a heterogeneous genetic contribution, with replicated associations at loci such as *APOE*, *GBA*, and *SNCA*, and suggests that part of the risk is linked to convergent pathways involving lipid and endo-lysosomal metabolism and protein homeostasis relevant to *α*-synuclein pathology (10, 11).

DLB thus emerges as a heterogeneous neurodegenerative entity in which α-synuclein pathology results from the convergence of intrinsic biological processes and external modulators across the lifespan. Nevertheless, despite the expanding literature on synucleinopathies, the genetic determinants and environmental factors specifically implicated in DLB are often extrapolated from PD, reflecting the relative scarcity of DLB-focused studies. While models originally developed in PD have provided important mechanistic insights that may apply to DLB, further expansion of disease-specific evidence is needed to refine these interpretations. In this context, the present review aims to integrate recent evidence on genetic susceptibility and environmental and metabolic factors involved in DLB, with particular emphasis on the pathophysiological mechanisms that link these domains and contribute to disease expression, focusing on studies specifically addressing DLB as well as those referring to Lewy body dementia or LBD when directly relevant.

## 2 Methods

This review was conducted using a narrative approach and did not follow a formal systematic review protocol. However, a structured search and selection process was applied to enhance transparency, without quantitative synthesis.

A literature search was conducted to identify relevant studies addressing genetic vulnerability and environmental factors in DLB. The databases searched included PubMed/MEDLINE, Scopus and Google Scholar, covering publications from January 2021 to January 2026.

Search terms were combined using Boolean operators and included “dementia with Lewy bodies” OR “Lewy body dementia” OR “Lewy body disease” together with “risk factor” OR “environmental exposure,” OR “environmental risk factors” OR “toxic exposure” OR “gut-brain axis.” Broader terms such as “alpha-synuclein” and “synucleinopathies” were initially explored; however, these yielded predominantly literature focused on PD without specific analysis of DLB and were therefore excluded from the final search strategy.

No initial restrictions were applied regarding study design or sample size. Studies were included if they: (1) specifically addressed DLB, Lewy body dementia, or LBD, (2) evaluated genetic, environmental, or metabolic factors, and (3) were published in English. Studies were excluded if they (1) focused exclusively on PD without clear relevance to DLB, (2) did not contribute directly to the conceptual scope of genetic and environmental mechanisms.

The search identified 111 records in PubMed, 592 in Scopus, and a large number of results in Google Scholar, from which the first 200 entries sorted by relevance were screened. Duplicates were removed. Original research articles were prioritized to construct the core of the review. The final selection included 26 original studies and 2 systematic reviews. Additional narrative reviews were incorporated to support conceptual synthesis and provide context.

## 3 Biological susceptibility in dementia with Lewy bodies: genetic determinants and environmental factors

**Figure 1:**
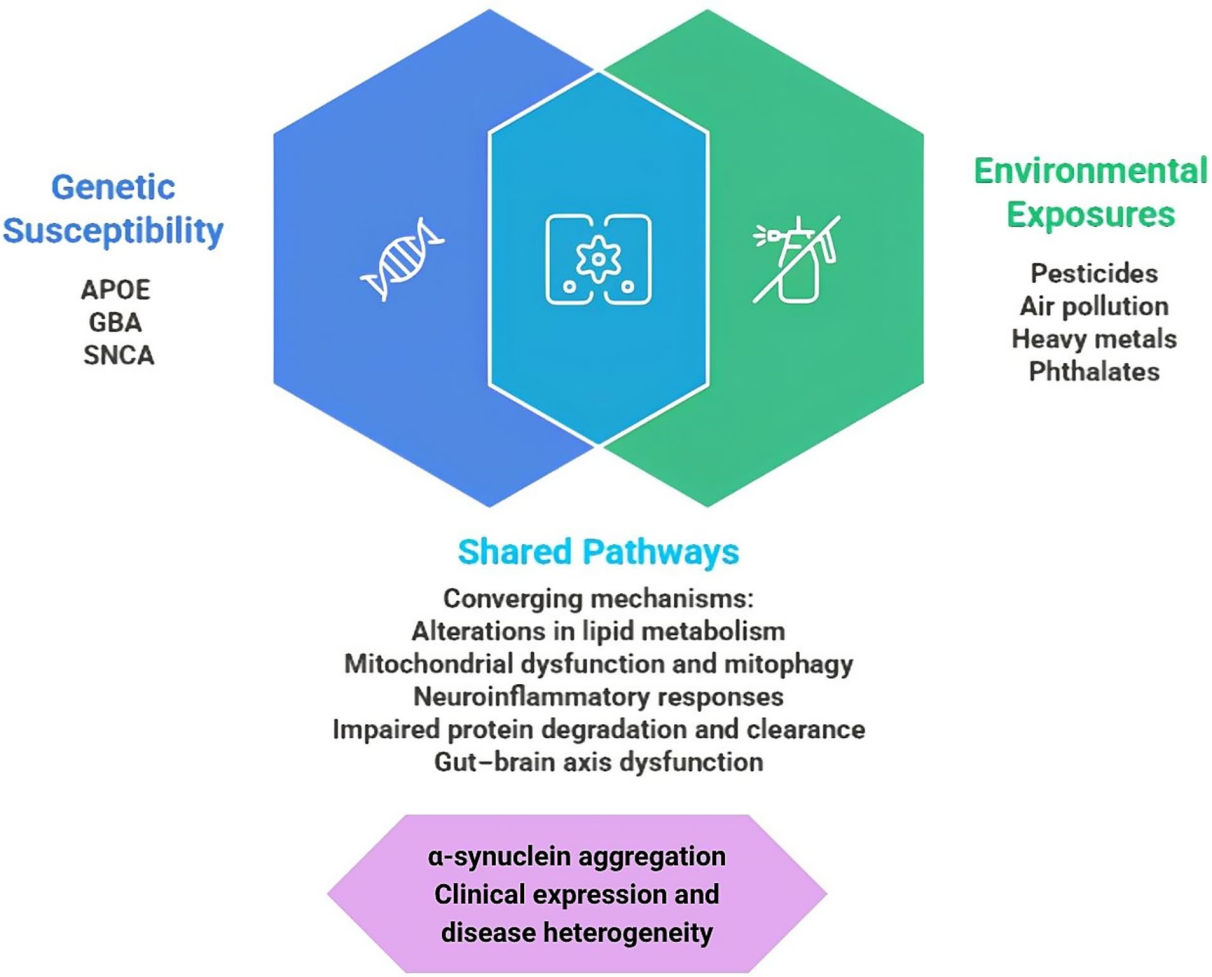
Conceptual framework of genetic and environmental contributions to dementia with Lewy bodies. Genetic susceptibility and environmental exposures converge on shared biological pathways and may promote *α*-synuclein aggregation, contributing to clinical expression and disease heterogeneity. Created with Napkin AI and Canva based on the authors’ specifications.

A single causal factor cannot explain susceptibility to DLB; rather, it may arise from the convergence of genetic determinants and environmental exposures on biologically vulnerable systems (Figure 1). In this section, we first review major genetic determinants and pathophysiological mechanisms implicated in DLB and subsequently examine environmental and metabolic factors that interact with this biological susceptibility, with particular emphasis on toxic exposures, metabolic disruption, and gut–brain axis.

### 3.1 Genetic determinants and pathophysiological mechanisms in dementia with Lewy bodies

Genetic susceptibility to DLB is characterized by moderate heritability, recently estimated at approximately 34% through extended family analyses. This genetic background does not act in a deterministic manner but instead contributes to a biologically vulnerable substrate marked by substantial heterogeneity, with genetic risk operating in concert with additional modifiers across the lifespan (12). Clustering analyses have identified patient subgroups with a low burden of known common risk variants, suggesting that, in some cases, alternative pathogenic routes or greater influence of environmental and epigenetic factors may be present (13).

#### 3.1.1 Major genetic axes: *APOE*, *GBA*, and *SNCA*

Principal genetic contributors delineate distinct neuropathological trajectories within the disease spectrum. APOE ε4 allele, a key regulator of lipid transport associated with amyloid metabolism, represents the most robust risk factor for cases with significant amyloid copathology (DLB–AD subgroup). GBA E326K variant, encoding lysosomal enzyme glucocerebrosidase, is more strongly associated with “pure” DLB phenotypes and earlier age at onset (13, 14). In contrast, SNCA, encoding *α*-synuclein, a key protein in Lewy body pathology, may modulate susceptibility through variation in gene dosage and regulatory elements. Spatial transcriptomic studies have shown that increased SNCA expression is concentrated in specific cortical layers, particularly layer V, which exhibits the highest Lewy body burden and marked metabolic vulnerability (14, 15).

#### 3.1.2 Lipid biology and intercellular propagation

Multiple lines of evidence suggest that lipid metabolism is a central mediator of pathogenesis. Deficiency of lysosomal enzyme glucocerebrosidase (GCase), encoded by *GBA*, has been associated with ceramide accumulation packaged into extracellular vesicles (EVs) (16). These EVs, involved in cell-to-cell communication, may act as pathological carriers facilitating intercellular propagation and aggregation of wild-type *α*-synuclein, a process occurring independently of *GBA* mutational status in some models (16, 17). In addition, loss of APOE function could exacerbate lysosomal dysfunction, which is associated with GBA reduction and accumulation of insoluble *α*-synuclein (18).

#### 3.1.3 Failure of mitochondrial quality control

Mitochondrial dysfunction has been implicated in LBD. In cortical tissue, reduced mitochondrial DNA copy number and decreased expression of mitochondrial biogenesis regulators have been reported, with more pronounced changes in DLB (19). Mechanistically, deficiency of the mitochondrial kinase PINK1, a key regulator of mitophagy (selective degradation of damaged mitochondria), may lower the threshold for *β*-amyloid–induced spontaneous *α*-synuclein aggregation, creating a permissive environment for the development of cortical pathology (20). Furthermore, the mitochondrial damage marker pS65-Ub is increased in APOE ε4 carriers with LBD, linking genetic background to alterations in mitochondrial quality control and mitophagy (21).

#### 3.1.4 Neuroinflammation and epigenetic regulation

Accumulation of lipid substrates and misfolded proteins may contribute to chronic immune activation in LBD. In models of GCase deficiency, accumulation of the lipid substrate glucosylceramide promotes activation of macrophages, which in turn could contribute to the development of neuropathology beyond the direct consequences of lysosomal dysfunction (22).

Epitranscriptomic mechanisms may further modulate neuroinflammatory responses. The N6-methyladenosine (m6A) RNA methyltransferase METTL3, a key component of the m6A “writer” complex responsible for RNA methylation, is upregulated in synucleinopathies, including DLB. Experimental studies show that METTL3 and its downstream m6A readers can regulate the pro-inflammatory secretome of activated microglia exposed to *α*-synuclein fibrils, suggesting that RNA methylation pathways may contribute to sustained neuroinflammatory signaling in these disorders (23).

Finally, epigenetic alterations at the DNA level have also been described in DLB. Genome-wide DNA methylation analyses of post-mortem brain tissue have identified differentially methylated CpG sites and regions, including loci within genes such as PAK6 and LIPA, indicating that DNA methylation changes may represent an additional layer of molecular regulation contributing to DLB pathogenesis (24).

#### 3.1.5 Additional factors: protection versus risk

Genetic variation also reveals protective mechanisms, such as *APOE3-Jacksonville* (V236E) variant, which may reduce DLB risk by decreasing apoE aggregation and improving lipidation (25). Conversely, sex-specific risk factors have been identified, including *MAP3K15* locus on X chromosome, which preferentially affects women (26).

In summary, available evidence suggests that genetic susceptibility in DLB does not converge on a single pathogenic axis. Rather, genetic determinants establish a predisposed biological landscape upon which additional modulators may influence the neurodegenerative process. However, much of the available evidence derives from experimental models and postmortem or genetic association studies, often involving limited sample sizes or rare variants, which should be considered when interpreting mechanistic inferences. This framework provides a basis for analysis of non-genetic factors addressed in the following section.

### 3.2 Environmental and metabolic factors in the pathogenesis of dementia with Lewy bodies

Etiology of DLB likely reflects a complex interplay of genetic susceptibility and environmental exposures. Evidence from LBD suggests that environmental factors may account for a substantial proportion of cases (27). It is proposed that these exposures do not act in isolation but instead converge on biological systems that modulate *α*-synuclein aggregation, neuroinflammation, and mitochondrial dysfunction (28). In this context, gut–brain axis mechanisms may represent a critical interface through which peripheral toxicants and intestinal pathophysiology could contribute to neurodegenerative processes relevant to LBD (27, 28).

The evidence supporting these factors is heterogeneous, ranging from epidemiological associations in human populations to mechanistic insights derived from experimental models, which should be interpreted accordingly.

#### 3.2.1 Pesticides and protein degradation pathways: the paraquat model

Exposure to the herbicide paraquat (PQ) represents one of the most consistently documented environmental risk factors for LBD, showing a clear dose–response relationship (27). Experimental models demonstrate that PQ not only induces *α*-synuclein inclusion formation but also impairs its efficient clearance by disrupting the aggresome–autophagy–lysosome pathway. Specifically, PQ may induce upregulation of histone deacetylase 6 and downregulation of dynein, thereby impairing transport and degradation of *α*-synuclein aggregates toward microtubule-organizing centers (29). In addition, PQ exposure has been associated with early intestinal dysfunction, characterized by colonic inflammation and dysbiosis, which precedes central nervous system pathology, suggesting a “body-first” pattern of disease propagation (27).

It should be considered that most evidence regarding paraquat exposure is derived from experimental models, and its direct contribution to DLB in humans remains uncertain.

#### 3.2.2 Air pollution and induction of pathogenic strains

Ambient air pollution has recently been postulated as a latent driving force of dementia, acting as a critical modifiable risk factor that induces neuroinflammatory and structural brain changes decades before clinical disease onset (30).

Within the spectrum of Lewy body dementia, epidemiological evidence derived from cohorts exceeding 50 million individuals has linked chronic exposure to fine particulate matter (PM2.5) with a significantly increased risk of first hospitalization (31).

At a mechanistic level, PM2.5 may act as a potent catalyst of *α*-synuclein aggregation and also promote the formation of a highly pathogenic and neurotoxic *α*-synuclein strain, termed PM-PFF. This strain exhibits increased resistance to proteinase K degradation and enhanced propagation capacity, closely resembling α-synuclein strains amplified from patients with Lewy body dementia, including DLB. Experimental studies further demonstrate that inhalation of PM2.5 induces cerebral atrophy, particularly affecting the hippocampus and medial temporal lobe, as well as progressive axonal damage. Notably, these neurodegenerative changes and associated cognitive deficits are markedly attenuated in *α*-synuclein–deficient models, suggesting a central role for α-synuclein in pollution-induced neurotoxicity (31).

Beyond direct effects on α-synuclein pathology, air pollution may further influence the clinical expression of DLB by exacerbating mixed neuropathological profiles. In aging brains, PM2.5 exposure has been shown to increase Alzheimer-type neuropathology, including amyloid plaques and tau tangles, which in turn may mediate dementia severity (32). Given the high prevalence of concomitant Alzheimer pathology in DLB, these findings suggest the notion that chronic exposure to air pollutants accelerates the convergence of multiple proteopathic pathways, thereby contributing to disease severity and progression in DLB (30, 32).

However, most of these findings derive from large-scale epidemiological associations or experimental models. Therefore, although air pollution is consistently linked to biological processes relevant to synucleinopathies, direct causal relationships and disease-specific effects in DLB remain unclear.

#### 3.2.3 Heavy metals and cumulative neurotoxicity

Disruption of metal homeostasis, particularly involving lead and copper, may represent another environmental factor relevant to LBD. Chronic or intermittent lead exposure increases both the number and size of *α*-synuclein inclusions, leading to axonal damage and long-term memory deficits in experimental models of synucleinopathies (33). Copper has also been proposed as a potent promoter of α-synuclein oligomerization and fibrillation. In a single case report, prolonged ingestion of copper-contaminated drinking water was associated with early-onset DLB, suggesting that excessive copper levels may act as a disease modifier in genetically susceptible individuals (34).

Overall, the evidence reviewed is derived from experimental models or isolated clinical observations, which limits generalizability and precludes causal inference.

#### 3.2.4 Metabolic disruption and intestinal microbiome

Emerging evidence implicates phthalates (PAEs) and other endocrine-disrupting chemicals as modulators of clinical severity in DLB. Patients with DLB exhibit elevated urinary levels of phthalate metabolites, such as DEHP, which correlate with dementia severity and alterations in the intestinal metabolome, particularly bile acids and short-chain fatty acids (35). These compounds disrupt gut microbial composition, favoring genera such as *Collinsella* and *Ruminococcus torques*, which increase intestinal permeability and expose the enteric nervous system to endotoxins and xenobiotics (36). Enteroendocrine cells, which physiologically express *α*-synuclein, may function as luminal sensors of these toxicants, potentially facilitating protein misfolding and subsequent transfer to the vagus nerve (28).

Nevertheless, current evidence remains associative and derived from relatively small or cross-sectional studies and should therefore be interpreted with caution.

Overall, these findings across environmental and metabolic domains suggest that DLB arises from a convergence in which environmental toxicants exploit the vulnerability of protein quality control systems and biological barrier integrity, facilitating the formation and propagation of pathogenic *α*-synuclein strains from the periphery to the brain.

## 4 Discussion

The genetic architecture of DLB reveals a heterogeneous landscape of biological susceptibility, in which inherited risk does not operate as an isolated determinant but rather shapes specific neuropathological trajectories (3, 37). Convergence of variants in key genes such as *APOE* and *GBA* is not only associated with the presence of Alzheimer-type copathology or with earlier disease onset, but also impacts central homeostatic nodes, including lipid metabolism, mitochondrial quality control, and microglia-mediated immune activation (16, 20, 23, 38). These cellular pathways, further modulated by additional layers of complexity, such as epigenetic regulation and sex-dependent factors, including the MAP3K15 locus, help define the individual threshold for *α*-synuclein aggregation and propagation (22, 24, 26). Collectively, these findings support the notion that genetic determinants in DLB delineate distinct biological subgroups rather than a uniform continuum of risk (13, 25). Consistent with this, a recent study using CSF Aβ/tau and *α*-synuclein seed amplification assays in a cohort of 795 cognitively impaired individuals shows that coexisting Lewy body and Alzheimer-type pathology can be identified in 22.3% of individuals and is associated with accelerated cognitive decline compared to AD without Lewy body pathology (39).

In parallel, studies on environmental and metabolic factors indicate that exposure to pesticides, air pollutants, heavy metals, and endocrine-disrupting chemicals may activate or amplify pre-existing vulnerabilities, such as *α*-synuclein aggregation, mitochondrial dysfunction, and neuroinflammation (27, 31, 35). However, most of this evidence derives from experimental models, observational studies, or indirect mechanistic inferences. Consequently, although these exposures are consistently associated with biological alterations plausibly relevant to DLB, they do not allow direct causal relationships to be established, nor do they clearly discriminate whether such factors act as initial triggers or as accelerators of an already established pathological process (30, 32).

Juxtaposition of the genetic and environmental domains highlights points of convergence rather than formally demonstrated interactions. Most available evidence reflects parallel contributions of genetic susceptibility and environmental exposures converging on shared biological pathways, rather than studies explicitly demonstrating interaction effects. Recurrently, protein quality control systems, lipid metabolism, mitophagy, and biological barrier integrity emerge as shared nodes at which genetic variants and toxic exposures intersect (16, 21, 28). Nevertheless, the studies reviewed rarely evaluate both components simultaneously in well-characterized DLB cohorts, limiting the ability to infer true synergistic or additive effects.

Beyond mechanistic insights, these conceptual considerations may have implications for how DLB is understood in clinical and population-based contexts. From a clinical perspective, this framework suggests that DLB may be conceptualized as a disorder of differential vulnerability, in which multiple biological systems may be selectively affected. Nonetheless, translation of these mechanisms into clinical practice remains limited. From an epidemiological perspective, evidence supporting modifiable risk factors in DLB remains limited and heterogeneous. Most studies address broader dementia or synucleinopathy outcomes, constraining disease-specific inference. However, the identification of environmental exposures as potential modulators highlights a possible avenue for prevention, although specific strategies cannot yet be defined with certainty.

This review has limitations inherent to its narrative design and to its focus on literature published over the past 5 years. The clinical and neuropathological heterogeneity of DLB, together with frequent extrapolation from other synucleinopathies, constrains the availability of disease-specific evidence. Many environmental studies lack neuropathological confirmation and are based on observational or indirect evidence, often without detailed genetic stratification, whereas genetic studies also lack precise information regarding lifetime environmental exposures.

In this context, DLB emerges less as a disorder defined by a single pathogenic cascade and more as a condition shaped by differential susceptibility across individuals and across the lifespan. Current evidence supports a framework in which genetic background constrains the biological systems most vulnerable to perturbation, while environmental and metabolic exposures modulate the timing, severity, and anatomical expression of pathology. However, the absence of studies that systematically integrate these dimensions in DLB-specific cohorts represents a critical gap. Addressing this gap will require longitudinal designs that move beyond extrapolation from related synucleinopathies and incorporate genetic stratification, quantified lifetime exposures, and multimodal biomarkers. Such an approach is essential to refine disease models, improve patient stratification, and ultimately shift the field toward mechanism-informed prevention and intervention strategies tailored to DLB.
